# A dual inhibitor overcomes drug-resistant FLT3-ITD acute myeloid leukemia

**DOI:** 10.1186/s13045-021-01098-y

**Published:** 2021-07-03

**Authors:** Peihong Wang, Xinhua Xiao, Yuyin Zhang, Baoyuan Zhang, Donghe Li, Mingzhu Liu, Xi Xie, Chenxuan Liu, Ping Liu, Ruibao Ren

**Affiliations:** Shanghai Institute of Hematology, State Key Laboratory for Medical Genomics, National Research Center for Translational Medicine (Shanghai), International Center for Aging and Cancer, Collaborative Innovation Center of Hematology, Ruijin Hospital, Shanghai Jiao Tong University School of Medicine, Shanghai, China

**Keywords:** Acute myeloid leukemia, FLT3-ITD, FLT3 resistance mutation, KX2-391, AC220

## Abstract

FLT3 mutations are the most frequently identified genetic alterations in acute myeloid leukemia (AML) and are associated with poor prognosis. Multiple FLT3 inhibitors are in various stages of clinical evaluation. However, resistance to FLT3 inhibitors resulting from acquired point mutations in tyrosine kinase domain (TKD) have limited the sustained efficacy of treatments, and a “gatekeeper” mutation (F691L) is resistant to most available FLT3 inhibitors. Thus, new FLT3 inhibitors against both FLT3 internal tandem duplication (FLT3-ITD) and FLT3-TKD mutations (including F691L) are urgently sought. Herein, we identified KX2-391 as a dual FLT3 and tubulin inhibitor and investigated its efficacy and mechanisms in overcoming drug-resistant FLT3-ITD-TKD mutations in AML. KX2-391 exhibited potent growth inhibitory and apoptosis promoting effects on diverse AML cell lines harboring FLT3-ITD mutations and AC220-resistant mutations at the D835 and F691 residues in TKD and inhibited FLT3 phosphorylation and its downstream signaling targets. Orally administered KX2-391 significantly prolonged the survival of a murine leukemia model induced by FLT3-ITD-F691L. KX2-391 also significantly inhibited the growth of 4 primary AML cells expressing FLT3-ITD and 2 primary AML cells expressing FLT3-ITD-D835Y. Our preclinical data highlight KX2-391 as a promising FLT3 inhibitor for the treatment of AML patients harboring FLT3 mutations, especially refractory/relapsed patients with F691L and other FLT3-TKD mutations.

**Supplementary Information:**

The online version contains supplementary material available at 10.1186/s13045-021-01098-y.

To the editor

FLT3 mutations occur in more than 30% of patients with acute myeloid leukemia (AML) and are associated with short relapse-free and overall survival, including internal tandem duplication (ITD) and point mutations within the tyrosine kinase domain (TKD) [1, 2]. To date, multiple FLT3 kinase inhibitors have been developed and some are approved for clinical use including sorafenib, Quizartinib (AC220) and Gilteritinib [3]. However, clinical responses to these drugs are transient because of high rates of relapse and drug resistance after treatment, which contributes to disease progression and poor overall survival [4, 5]. One particular mechanism for resistance involves acquired additional mutations in the TKD and a “gatekeeper” mutation (F691L) is resistant to most currently available FLT3 inhibitors [6, 7]. Therefore, finding effective compounds to overcome the drug resistance caused by F691L and other mutations is an urgent problem. Herein, we identified KX2-391 as a FLT3 inhibitor and evaluated its activity against FLT3-ITD-TKD mutations using in vitro and in vivo models.

We used molecular docking simulations to computationally screen 1487 small molecule ligands from the L3400 Clinical Compound Library and identified KX2-391 as a candidate FLT3 inhibitor (Additional file 1: Table S1). Previous studies reported KX2-391 was an SRC/tubulin dual inhibitor and exhibited anticancer activities in some tumors [8]. Our modeling predicted the interaction of KX2-391 with FLT3’s L616, V624 and E661 residues (Additional file 2: Fig. S1). We further confirmed the interactions between FLT3 and KX2-391 by cellular thermal shift assay. Compared with DMSO, an obvious thermal shift of the melting curve was detected in the KX2-391-treated sample. The thermal stability of FLT3 protein was increased by KX2-391 in a dose-dependent manner (Fig. 1a). We evaluated the inhibitory activity of KX2-391 against different forms of FLT3 in Ba/F3 cells and found it potently inhibited the growth of FLT3-ITD-expressing Ba/F3 cells and all tested cells expressing FLT3-ITD-TKD mutations previously linked with drug resistance to FLT3 inhibitors, such as AC220 (Fig. 1b, c, Additional file 1: Table S2). Notably, Ba/F3-ITD-F691L cells were tenfold more sensitive to KX2-391 than parental Ba/F3 cells (0.032 μM vs 0.372 μM). KX2-391 also displayed higher inhibitory efficacy on human leukemia cell lines harboring FLT3-ITD (MV4-11 and MOLM13) than that on FLT3 nonmutated leukemia cell lines (Fig. 1d). We observed dose-dependent induction of apoptosis in Ba/F3 cells expressing FLT3-ITD, FLT3-ITD-D835Y and FLT3-ITD-F691L as well as in two FLT3-ITD positive AML cell lines (Fig. 1e, f). KX2-391 prominently inhibited the phosphorylation of FLT3 and downstream targets STAT5, ERK and AKT in FLT3-ITD, FLT3-ITD-F691L-expresssing Ba/F3 cells and other cells of our assay panel (Fig. 1g, h). Recalling that KX2-391 has to date understood as an SRC/tubulin inhibitor [9], we monitored SRC phosphorylation and assessed KX2-391’s effects on microtubule morphology. KX2-391 treatment did not alter phosphorylation of SRC in FLT3 mutant cells (Fig. 1g, h). We did detect disrupted tubulin polymerization in MOLM13 cells treated with KX2-391 and with various known tubulin inhibitors (Additional file 2: Fig. S2). Excluding a direct impact of tubulin modulation, assays showed that the known tubulin inhibitor Vincristine did not affect phosphorylation of FLT3 or its downstream targets (Additional file 2: Fig. S3).

**Fig. 1:**
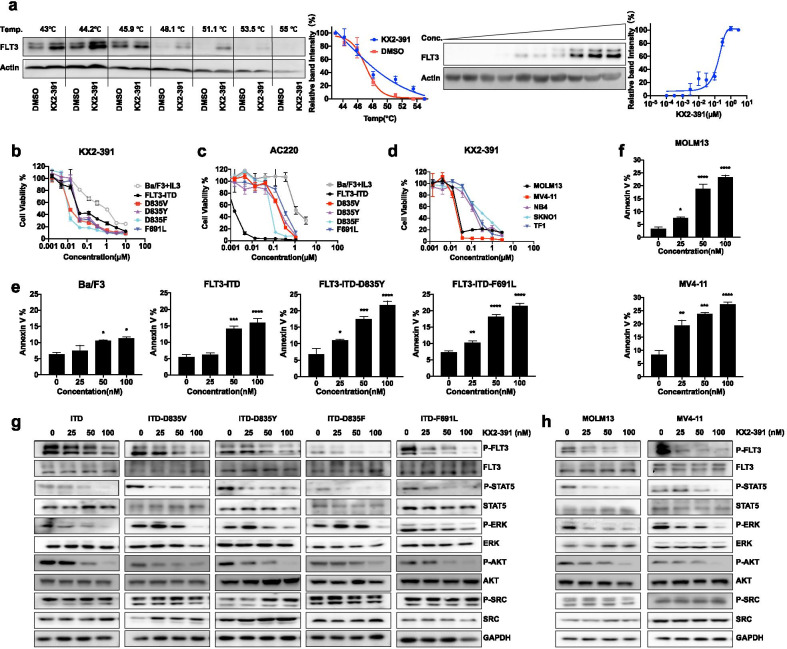
KX2-391 is active against ITD-TKD resistance-causing FLT3 mutations and blocks FLT3 signaling in FLT3-ITD and FLT3-ITD-TKD cells. **a** Quantification of cellular thermal shift assay was made using western blot Ba/F3 cells expressing FLT3-ITD cells were treated with KX2-391 (1 µM) for 1 h, and temperatures between 43 and 55℃ were defined to perform the test. KX2-391were treated based on 10 different concentrations for 1 h at 51℃. Data were normalized by setting the highest and lowest value in each set to 100% and 0%, respectively. Data were obtained in the presence of the KX2-391 (blue circle) as the positive control and DMSO (red square) as the negative control. **b** Normalized cell viability of Ba/F3 cells expressing FLT3-ITD TKD mutations after a 48 h exposure to various concentrations of KX2-391 and AC220 (**c**), measured using CellTiter Glo assays (error bars represent the SD of 3 or more independent experiments). **d** Viability of FLT3-ITD mutated cells (MV4-11, MOLM-13), and FLT3 nonmutated cells (NB4, SKNO1, TF1) treated with various concentrations of KX2-391 for 48 h, measured by CellTiter Glo assays. **e** Ba/F3 cells expressing FLT3-ITD TKD mutations were treated with different concentrations of KX2-391 for 24 h and then parental Ba/F + IL3, FLT3-ITD, FLT3-ITD-D835Y, and FLT3-ITD-F691L cells, as well as **f** MOLM13 and MV4-11 cells were examined by flow cytometry (Annexin V). **g** Ba/F3 cells expressing FLT3-ITD, FLT3-ITD-D835Y, FLT3-ITD/F691L, FLT3-D835V, and FLT3-D835F, as well as FLT3-ITD positive human leukemia cell lines **h** MOLM13 and MV4-11 were incubated for 12 h with the indicated concentrations of KX2-391 (based on the IC50 values) and subsequently examined by western blotting using antibodies against FLT3/P-FLT3, STAT5/P-STAT5, ERK/P-ERK, AKT/P-AKT and SRC/P-SRC. GAPDH was used as a loading control. All values represent the mean ± SD of three independent experiments. *P < 0.05; **P < 0.01; ***P < 0.001; ****P < 0.0001versus the control

Next, we used the previously described Ba/F3-ITD-F691L leukemia model to measure the efficacy of KX2-391 in vivo [10]. KX2-391 showed a significant benefit on median survival, prolonging the survival period from 12 days in the control group to 23.5 days (Fig. 2a, P < 0.0001). No significant weight loss (Fig. 2b) or other signs of toxicity were observed during treatment. Compared to the vehicle control, AC220 and Gilteritinib groups, KX2-391-treated mice had fewer leukemia cells (in peripheral blood, spleen, and bone marrow) (Fig. 2c, d) and had smaller spleens (Fig. 2d). H&E staining demonstrated that KX2-391 treatment significantly reduced AML cell infiltration into the spleen and liver (Fig. 2e).

**Fig. 2:**
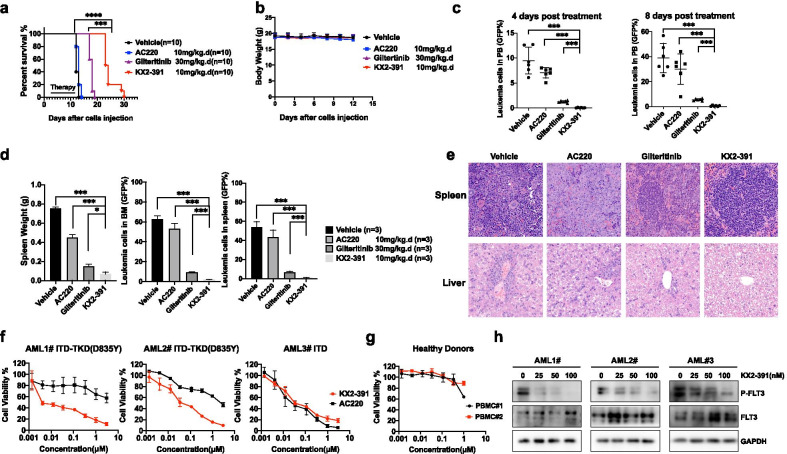
In vivo effects of KX2-391 in mice bearing FLT3-ITD-F691L leukemia and against patient leukemic blast cells harboring FLT3-ITD or FLT3-ITD-D835Y mutations. **a** Kaplan–Meier survival curves of FLT3-ITD-F691L leukemia mice administered vehicle, Gilteritinib (30 mg/kg), AC220 (10 mg/kg), or KX2-391 (10 mg/kg) once daily for 10 days (orally). **** *P* < 0.0001. **b** No change in body weight was detected between the KX2-391 treatment and vehicle control groups. **c** The percentage of GFP-positive Ba/F3 FLT3-ITD-F691L leukemia cells in peripheral blood (PB) samples from mice treated with vehicle, Gilteritinib (30 mg/kg/d), AC220 (10 mg/kg/d), or KX2-391 (10 mg/kg/d) for 4 days and for 8 days. **d** Representative weight of spleens at 10 days after injection with Ba/F3 FLT3-ITD-F691L leukemia cells, and Flow cytometry analysis of bone marrow cells and spleens from mice as described in (**c**). **e** Hematoxylin and eosin staining of spleens and liver from mice treated as described in (**a**). Scale bars in the panel are 50 μm. **f** Patient-derived AML leukemic blast cells expressing FLT3-ITD/D835Y (patients 1 and 2), FLT3-ITD (patient 3) and peripheral blood mononuclear cells (PBMCs) from healthy donors were incubated for 48 h with the indicated concentrations of KX2-391 (**g**), and the cell viability was then determined. For each FLT3 inhibitor, the percentage over DMSO control was presented as a mean value, with error bars representing ± SD. **h** KX2-391 suppresses FLT3 phosphorylation in primary AML cells incubated for 12 h with the indicated concentrations (based on the IC50 values), as determined by western blotting using the indicated antibodies. GAPDH was used as a loading control. ****P < 0.0001

We also evaluated the anti-leukemia effects of KX2-391 on primary AML cells isolated from 6 newly diagnosed FLT3-ITD AML patients (Additional file 1: Table S3). KX2-391 effectively reduced cell viability in 4 primary blasts with FLT3-ITD mutations (comparable with AC220’s effect). It also significantly inhibited the growth of 2 primary AML cells expressing FLT3-ITD-D835Y—both of which were resistant to AC220 (Fig. 2f, Additional file 2: Fig. S4). KX2-391 did not affect the growth of healthy peripheral blood mononuclear cells at the same concentrations (Fig. 2g). We confirmed that KX2-391 inhibited FLT3 phosphorylation in primary AML cells (Fig. 2h).

Collectively, we show that KX2-391 is an FLT3/tubulin inhibitor that exerts strong therapeutic effects both in vitro and in vivo against FLT3-ITD and drug resistant TKD mutations including FLT3-ITD-F691L, which is understood as the most difficult mutation to overcome clinically. Considering the detected therapeutic effects and its apparently minimal toxicity [11, 12], KX2-391 may become a useful second-line drug suitable for treating some of the most clinically challenging AML cases.

## Supplementary Information

**Additional file 1: Table S1**. 14 small molecule inhibitors are in clinical trials of leukemia. **Table S2**. Inhibitory activity of KX2-391 against FLT3 mutant and FLT3 nonmutated cell lines. **Table S3**. Patient information (DOCX 18 kb)

**Additional file 2: Figure S1**. Computational modeling of KX2-391 binding with wild-type FLT3. (a) An overview of the docking results of KX2-391 with FLT3 (Protein Data Bank: 5X02); two orthogonal views are shown. (b) Close-up of the KX2-391-FLT3 model, highlighting the hydrogen bond formed by residues LEU-616 and GLU-661, the predicted KX2-391 binding site are not included Phe691. **Figure S2**. KX2-391 disrupted microtubules in MOLM13 cells. MOLM13 cells were treated with dimethyl sulfoxide (DMSO, control), 100 nM Paclitaxel, 100 nM Vincristine, 100 nM Colchicine, and 100 or 200 nM KX2-391 for 4 h, and confocal microscopy was used to observe the signal corresponding to α-tubulin (green); DNA was counterstained with DAPI (blue). **Figure S3**. Vincristine (VCR) does not inhibit FLT3 signaling in FLT3-ITD or FLT3-ITD-TKD cells. Ba/F3 cells expressing FLT3-ITD, FLT3-ITD-D835Y, or FLT3-ITD-F691L, and human cell lines MOLM13 and MV4-11were incubated for 12 h with the indicated concentrations of VCR (based on the IC50 values) and subsequently examined by western blotting using the indicated antibodies. GAPDH was used as a loading control. **Figure S4**. Potent inhibition of KX2-391 against patient leukemic blast cells harboring FLT3-ITD. Patient-derived AML leukemic blast cells expressing FLT3-ITD (patients 4-6) were incubated for 48 h with the indicated concentrations of KX2-391 and AC220, and the cell viability of blast cells was then determined with CellTiter Glo assays. For each FLT3 inhibitor, the percentage over DMSO control was presented as a mean value, with error bars representing ±SD.

## Data Availability

All data generated or analyzed during this study are included in this published article and its supplementary information files.

## Abbreviations

AML: Acute myelogenous leukemia
BMCs: Bone marrow cells
FLT3: FMS-like tyrosine kinase 3
ITD: Internal tandem duplication
TKD: Tyrosine kinase domain
ERK: Extracellular signal regulated kinase
STAT: Signal transducer and activator of transcription
VCR: Vincristine
IC50: Half maximal inhibitory concentration
GFP: Green fluorescent protein
APC: Allophycocyanin
H&E: Hematoxylin and eosin
SDS: Sodium dodecyl sulfate
