# Supplementary material for: A dual inhibitor overcomes drug-resistant FLT3-ITD acute myeloid leukemia
Source: J Hematol Oncol. 2021 Jul 3;14:105. doi: 10.1186/s13045-021-01098-y (PMC8255005; doi:10.1186/s13045-021-01098-y)
Supplement: Supplementary file 1 — Additional file 1: Table S1. 14 small molecule inhibitors are in clinical trials of leukemia. Table S2. Inhibitory activity of KX2-391 against FLT3 mutant and FLT3 nonmutated cell lines. Table S3. Patient information (DOCX 18 kb) [file 13045_2021_1098_MOESM1_ESM.docx]

**Additional File1:**

**Table S1 14 small molecule inhibitors are in clinical trials of leukemia**

| Name | molecular weight | Affinity(kcal/mol) | Confirmed FLT3 target |
| --- | --- | --- | --- |
| KX2-391 | 431.5 | -9.8 | NO |
| Bemcentinib | 506.6 | -10.4 | NO |
| Idasanutlin | 616.5 | -11 | NO |
| Imatinib | 493.6 | -9.7 | NO |
| ICG001 | 548.6 | -10.7 | NO |
| LY2090314 | 512.5 | -10.4 | NO |
| KW2449 | 332.4 | -9.7 | YES |
| PLX51107 | 438.5 | -9.8 | YES |
| Linifanib | 375.4 | -10.1 | YES |
| Ponatinib | 532.6 | -11.3 | YES |
| Nilotinib | 529.5 | -10.8 | YES |
| Ibrutinib | 440.5 | -10.9 | YES |
| Sorafenib | 464.8 | -9.8 | YES |
| Crenolanib | 443.5 | -9.6 | YES |

**Table S2 Inhibitory activity of KX2-391 against FLT3 mutant cell lines**

| Cell line | IC50(nM) | |
| --- | --- | --- |
|  | AC220 | KX2-391 |
| Parental Ba/F3+IL3 | 884 | 372 |
| BaF3-FLT3-ITD | 1.38 | 19.5 |
| BaF3-FLT3-ITD-D835Y | 253.2 | 27.3 |
| BaF3-FLT3-ITD-D835V | 172.8 | 10.6 |
| BaF3-FLT3-ITD-D835F | 65.3 | 9.6 |
| BaF3-FLT3-ITD-F691L | 329.4 | 37.1 |
| MV4-11 | 3.5 | 14.2 |
| MOLM13 | 1.9 | 15.4 |
| SKNO1 | >1000 | 246.6 |
| TF1 | >1000 | 135.1 |
| NB4 | >1000 | 115.0 |
| Abbreviations: FLT3, Fms-like tyrosine kinase 3; IC50, the biochemical half maximal inhibitory concentration; ITD, internal tandem duplication | | |

**Table S3 Primary human FLT-ITD AML samples**

| ID sample | Age | Gender | Source | FAB subclass | FLT3 status | Other genetic abnormalities |
| --- | --- | --- | --- | --- | --- | --- |
| AML #1 | 55 | male | BM | M4 | ITD/TKD(D835Y) | Not found |
| AML #2 | 42 | female | BM | MDS | ITD/TKD(D835Y) | NPM1 |
| AML #3 | 44 | male | BM | M4 | ITD | CBFB-MYH1 |
| AML #4 | 56 | male | BM | M4 | ITD | MLL-PTD |
| AML #5 | 59 | male | BM | M5 | ITD | NPM1; DNMT3A |
| AML #6 | 58 | male | BM | M5 | ITD | NPM1 |
