# Supplementary material for: A dual inhibitor overcomes drug-resistant FLT3-ITD acute myeloid leukemia
Source: J Hematol Oncol. 2021 Jul 3;14:105. doi: 10.1186/s13045-021-01098-y (PMC8255005; doi:10.1186/s13045-021-01098-y)

**Supplementary Figures**

**Figure S1. Computational modeling of KX2-391 binding with wild-type FLT3.** (a) An overview of the docking results of KX2-391 with FLT3 (Protein Data Bank: 5X02); two orthogonal views are shown. (b) Close-up of the KX2-391-FLT3 model, highlighting the hydrogen bond formed by residues LEU-616 and GLU-661.

**Figure S2.** **KX2-391 disrupted microtubules in MOLM13 cells.** MOLM13 cells were treated with dimethyl sulfoxide (DMSO, control), 100 nM Paclitaxel, 100 nM Vincristine, 100 nM Colchicine, and 100 or 200 nM KX2-391 for 4 h, and confocal microscopy was used to observe the signal corresponding to α-tubulin (green); DNA was counterstained with DAPI (blue).

**Figure S3. Vincristine (VCR) does not inhibit FLT3 signaling in FLT3-ITD or FLT3-ITD-TKD cells.** Ba/F3 cells expressing FLT3-ITD, FLT3-ITD-D835Y, or FLT3-ITD-F691L and human leukemia cell lines MOLM13 and MV4-11were incubated for 12 h with the indicated concentrations of VCR (based on the IC50 values) and subsequently examined by western blotting using the indicated antibodies. GAPDH was used as a loading control.

**Figure S4. Potent inhibition of KX2-391 against patient leukemic blast cells harboring FLT3-ITD.** Patient-derived AML leukemic blast cells expressing FLT3-ITD (patients 4-6) were incubated for 48 h with the indicated concentrations of KX2-391 and AC220, and the cell viability of blast cells was then determined with CellTiter Glo assays. For each FLT3 inhibitor, the percentage over DMSO control was presented as a mean value, with error bars representing ±SD.


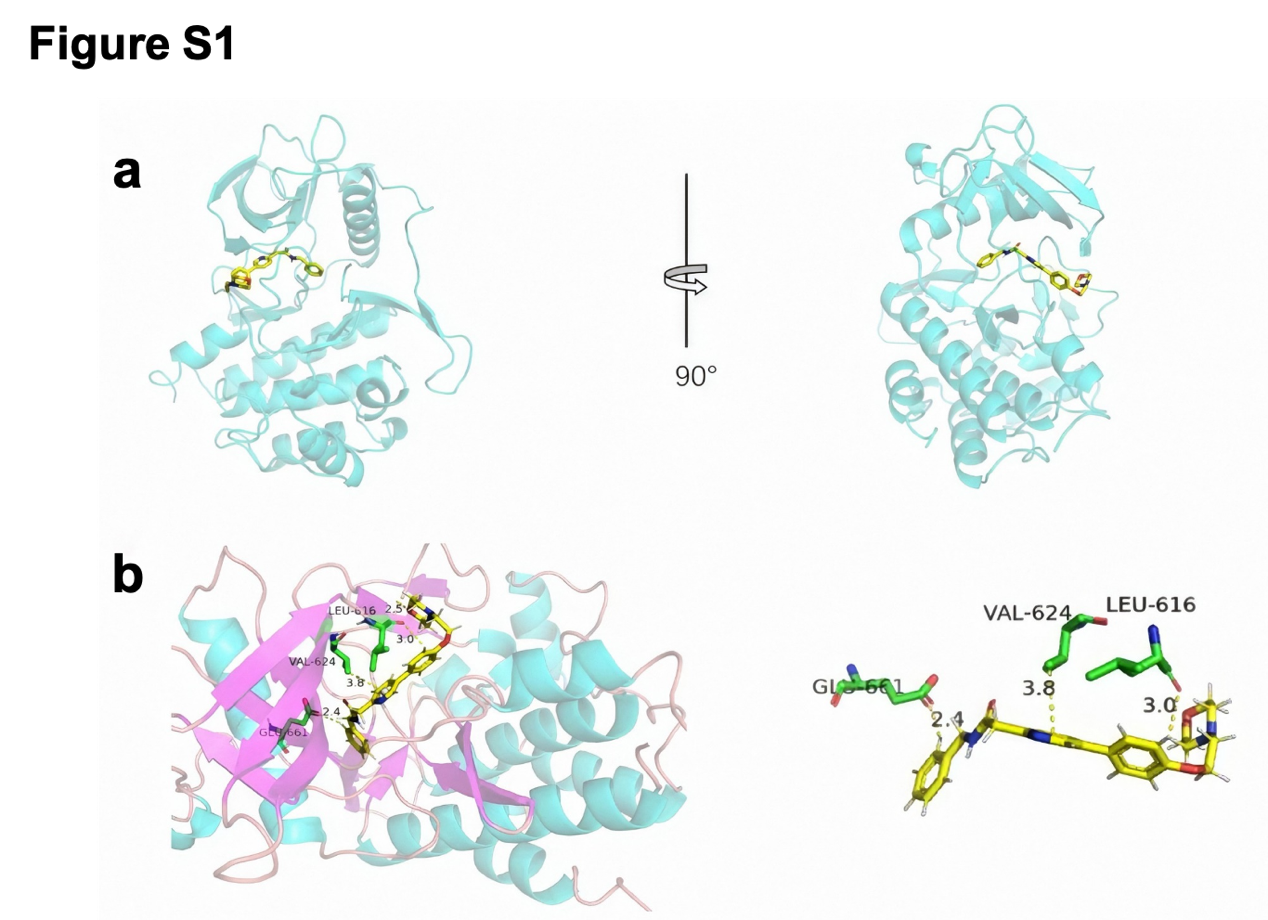

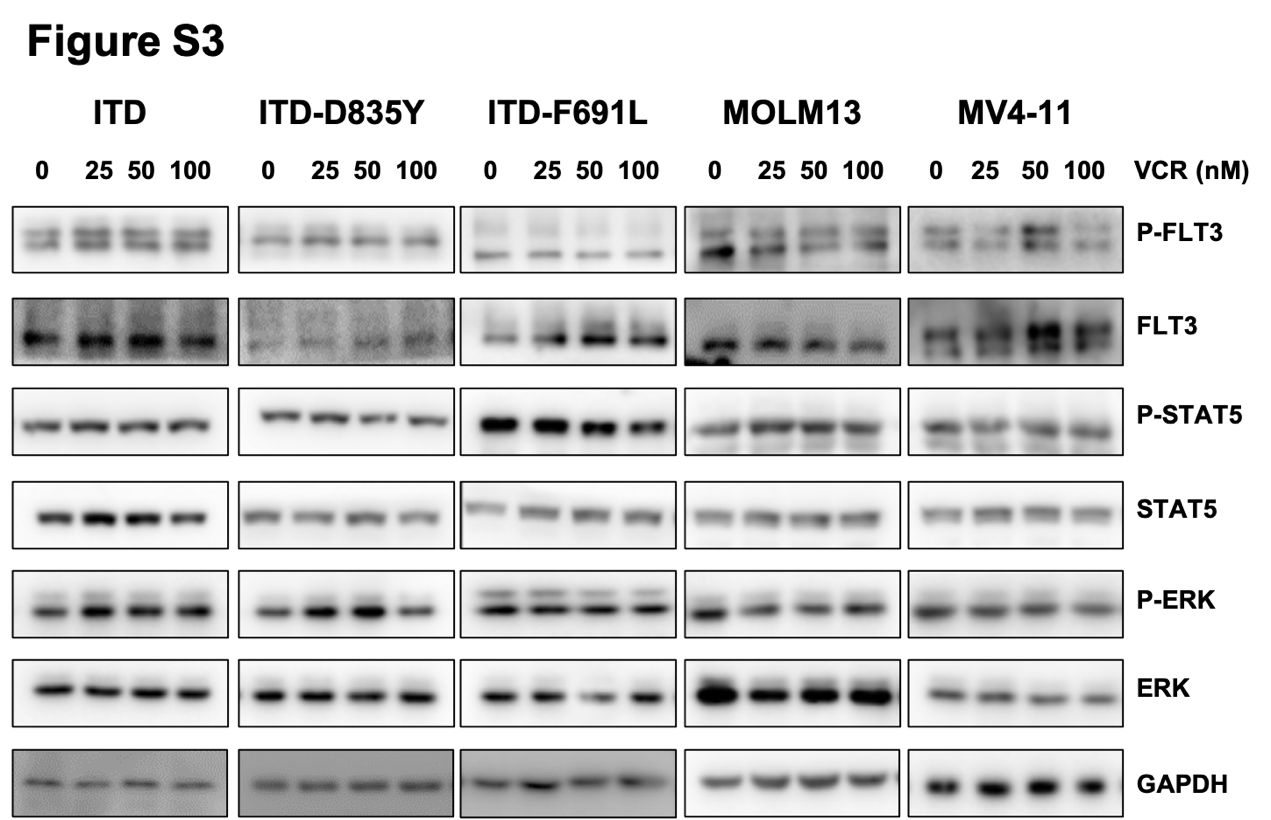


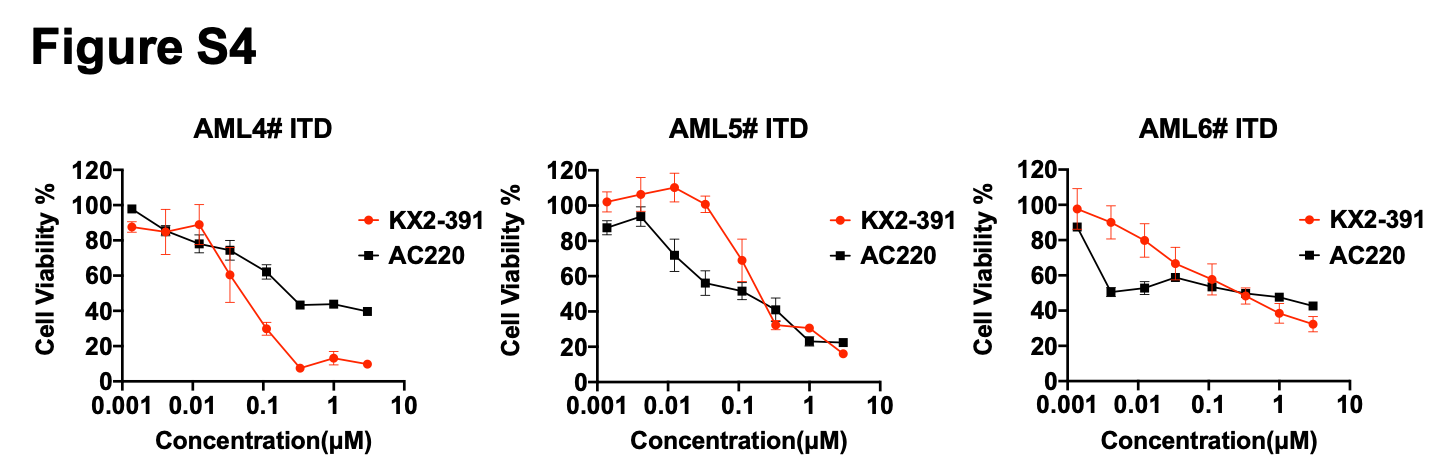

Supplement: Supplementary file 2 — Additional file 2: Figure S1. Computational modeling of KX2-391 binding with wild-type FLT3. (a) An overview of the docking results of KX2-391 with FLT3 (Protein Data Bank: 5X02); two orthogonal views are shown. (b) Close-up of the KX2-391-FLT3 model, highlighting the hydrogen bond formed by residues LEU-616 and GLU-661, the predicted KX2-391 binding site are not included Phe691. Figure S2. KX2-391 disrupted microtubules in MOLM13 cells. MOLM13 cells were treated with dimethyl sulfoxide (DMSO, control), 100 nM Paclitaxel, 100 nM Vincristine, 100 nM Colchicine, and 100 or 200 nM KX2-391 for 4 h, and confocal microscopy was used to observe the signal corresponding to α-tubulin (green); DNA was counterstained with DAPI (blue). Figure S3. Vincristine (VCR) does not inhibit FLT3 signaling in FLT3-ITD or FLT3-ITD-TKD cells. Ba/F3 cells expressing FLT3-ITD, FLT3-ITD-D835Y, or FLT3-ITD-F691L, and human cell lines MOLM13 and MV4-11were incubated for 12 h with the indicated concentrations of VCR (based on the IC50 values) and subsequently examined by western blotting using the indicated antibodies. GAPDH was used as a loading control. Figure S4. Potent inhibition of KX2-391 against patient leukemic blast cells harboring FLT3-ITD. Patient-derived AML leukemic blast cells expressing FLT3-ITD (patients 4-6) were incubated for 48 h with the indicated concentrations of KX2-391 and AC220, and the cell viability of blast cells was then determined with CellTiter Glo assays. For each FLT3 inhibitor, the percentage over DMSO control was presented as a mean value, with error bars representing ±SD. [file 13045_2021_1098_MOESM2_ESM.docx]
